# Reliability of length measurements collected by community nurses and health volunteers in rural growth monitoring and promotion services

**DOI:** 10.1186/s12913-018-2909-0

**Published:** 2018-02-17

**Authors:** Matilda E. Laar, Grace S. Marquis, Anna Lartey, Katherine Gray-Donald

**Affiliations:** 1 Department of Family and Consumer Sciences, University of Ghana, P O Box LG 91, Legon, Accra, Ghana; 2 School of Dietetics and Human Nutrition, McGill University, 21,111 Lakeshore Road, Ste. Anne de Bellevue, Montreal, Quebec, H9X 3V9 Canada; 3 Department of Nutrition and Food Science, University of Ghana, P O Box LG 134, Legon, Accra, Ghana

**Keywords:** Length, Growth monitoring and promotion, Measurement reliability, Community health nurse, Community health volunteer

## Abstract

**Background:**

Length measurements are important in growth, monitoring and promotion (GMP) for the surveillance of a child’s weight-for-length and length-for-age. These two indices provide an indication of a child’s risk of becoming wasted or stunted, and are more informative about a child’s growth than the widely used weight-for-age index (underweight). Although the introduction of length measurements in GMP is recommended by the World Health Organization, concerns about the reliability of length measurements collected in rural outreach settings have been expressed by stakeholders. Our aim was to describe the reliability and challenges associated with community health personnel measuring length for rural outreach GMP activities.

**Methods:**

Two reliability studies (A and B), using 10 children less than 24 months each, were conducted in the GMP services of a rural district in Ghana. Fifteen nurses and 15 health volunteers (HV) with no prior experience in length measurements were trained. Intra- and inter-observer technical error of measurement (TEM), average bias from expert anthropometrist, and coefficient of reliability (R) of length measurements were assessed and compared across sessions. Observations and interviews were used to understand the ability and experiences of health personnel with measuring length at outreach GMP.

**Results:**

Inter-observer TEM was larger than intra-observer TEM for both nurses and HV at both sessions and was unacceptably (compared to error standards) high in both groups at both time points. Average biases from expert’s measurements were within acceptable limits, however, both groups tended to underestimate length measurements. The R for lengths collected by nurses (92.3%) was higher at session B compared to that of HV (87.5%). Length measurements taken by nurses and HV, and those taken by an experienced anthropometrist at GMP sessions were of moderate agreement (kappa = 0.53, *p* < 0.0001).

**Conclusions:**

The reliability of length measurements improved after two refresher trainings for nurses but not for HV. In addition, length measurements taken during GMP sessions may be susceptible to errors due to overburdened health personnel and crowded GMP clinics. There is need for both pre- and in-service training of nurses and HV on length measurements and procedures to improve reliability of length measurements.

**Electronic supplementary material:**

The online version of this article (10.1186/s12913-018-2909-0) contains supplementary material, which is available to authorized users.

## Background

The primary objective of growth monitoring and promotion (GMP) is to provide a medium for health and nutrition surveillance of individual children to allow early detection and intervention of growth faltering when it can be easily reversed [1]. In 2003, a survey of the World Health Organization (WHO) member countries revealed that 50% of Latin America and the Caribbean countries compared to 9% of their African counterparts measured height/length as part of GMP [2]. The Ghana Health Service’s (GHS) monthly outreach GMP program reaches remote rural areas that would otherwise have no nutrition or primary health care services. GMP in this setting entails the weighing of a child, plotting of weight on a weight-for-age Z-score (WAZ) chart, and the interpretation of the child’s growth curve which serves as a guide for nutrition counseling. Growth faltering is defined as a flattening or the descent of a child’s WAZ curve compared to the WHO 2006 standard curves for two consecutive months. However, although the 2008 national wasting (weight-for-length/ height Z score (WLZ or WHZ) < − 2) prevalence in infancy (29%) and stunting (length/height-for-age Z score (LAZ or HAZ) < − 2) prevalence (17%) warrant attention, only underweight (WAZ < − 2) is regularly assessed in Ghana [3]. No provision exists in GMP services to measure length [4]. Weight-for-age cannot distinguish between short children with adequate body weight for their length (stunted but not wasted) and children thin for their length (wasted with or without stunting) [5]. Length measurements are necessary to monitor mild (− 2 ≤ WLZ < − 1) to moderate (− 3 ≤ WLZ < − 2) wasting which, once identified, can be reversed with timely effective counseling [6]. There is evidence suggesting that a child’s wasting status influences the rate of linear growth [7–10], and linear growth retardation is directly associated with an increased risk of disease [11], poor mental development [12] and reduced productivity in later life [13]. In addition, wasting is important for assessing nutritional status when exact ages of children are not known [2].

Despite offering benefits, monitoring length presents institutional difficulties posed by the lack of equipment or maintenance of available ones, feasibility of implementation and training of health personnel, and a tradition of measuring only weight in the Ghanaian health system culture [14, 15]. Unlike weight, accurate length measurement requires two people, one to help with positioning the child and the other to take the readings. At the level of the measurer/anthropometrist, monitoring length of children can be challenging due to the vulnerability of length measurements to errors. A significant problem is that of unreliability as a result of measurement error variance due to variability in measurements taken by observers. Imprecision and inaccuracy can be caused by inaccurate instruments (length board/stadiometer) or inadequate or improper training of measurers resulting in technical (positioning subject, reading) errors [16–18].

To monitor length as part of GMP, length measurements must be accurate and precise. Health personnel must be able to understand and interpret growth (weight-for-length and length-for-age) charts properly, identify the appropriate actions that need to be taken, and communicate effectively with caregivers [19–21]. Under- or over-estimation of the length of young children could lead to misclassification and misinterpretation of individual wasting (low WLZ) and stunting (low LAZ) risk. Missed growth faltering results in no growth promotion action and thus thwarts the purpose of GMP.

Our objective was to determine the reliability of length measurements, collected by nurses and health volunteers for standardization exercises and at GMP sessions, in the context of a one-year capacity building exercise in rural Ghana and document their experiences in using length measurements as part of outreach GMP services.

## Methods

### Study design and setting

As part of a one-year nutrition capacity-building exercise (June 2013 to June 2014), trainings on measuring length and plotting on WLZ charts were provided to community nurses and health volunteers in outreach GMP clinics of the Upper Manya Krobo district (UMKD), Ghana. In this setting, community nurses were primarily responsible for GMP clinics and were assisted by GHS-recognized health volunteers who were residents of the communities. The two-year pre-service training of community nurses covers multiple practical sessions on the measurement of weights and mid-upper arm circumference of children. No training on lengths is provided as length is not routinely monitored as part of the GHS GMP. HV may receive a short training in measuring, plotting, and interpretation of the weights of children or learn on-the-job from nurses. In this study, the nurses and HV were trained to measure length using the WHO measurement and standardization protocols for length [22]. All health personnel were trained to both measure and assist with the collection of length measurements. Initial training of community nurses and health volunteers and subsequent refresher trainings were provided at regular intervals throughout the year (Table 1). None of the health personnel had prior experience with collecting length measurements before this training. Anthropometry (length and weight) standardization sessions were conducted at the training sessions held three (session A) and nine months (session B) after the initial training session. Case observations of all nurses and health volunteers providing services at community-based GMP clinics were conducted by an observer experienced in anthropometric data collection after six months of using length measurements. At the end of a year of using length in outreach GMP clinics, community nurses and health volunteers were interviewed to explore their experiences with using length measurements as part of community-based GMP clinics. The sequence of data collection activities is provided in Table 1.

**Table 1:** Timeline for data collection for length reliability study in the Upper Manya Krobo district, Ghana

Item/Month(s)	1	2	3	4	5	6	7	8	9	10	11	12	13
Trainings for length measurements	√		√	√			√			√			
Capacity-building Intervention		√	√	√	√	√	√	√	√	√	√	√	√
Standardization A (15 nurses and 15 HV^a^)				√									
Standardization B (same group of 15 nurses and 15 HV)										√			
Observations: Independent group of trained HV and community nurses at GMP^b^ clinics							√	√	√	√	√	√	√
Interviews with trained HV and nurses													√

^a^HV: health volunteers

^b^GMP: Growth Monitoring and Promotion

### Data collection

#### Anthropometry standardization at training sessions

Fifteen nurses and 15 health volunteers provided complete length data at both standardization sessions and attended all refresher trainings. The Multicentre Growth Reference Study’s (MGRS) standardization method was used [22]. Ten children of ages between 0 and 24 months were recruited from communities with GMP clinics in close proximity to the research and training center. At each standardization session, the lengths of 10 children were measured, first by an experienced anthropometrist and an assistant, and then by a nurse or HV with the assistance of another nurse or HV, respectively. Length measurements were recorded to the nearest 0.5 cm using length boards (ADE GmbH & Co; Hamburg, Germany) and weights to the nearest 0.1 kg using Salter spring scales (Shorr production; Maryland, United States) provided to the GMP clinics. Nurses and health volunteers measured each child twice and were paired to assist each other with positioning for length measurements. For each of these health personnel, the first and second measurements of each child were separate (in time and on paper) to ensure independence. Using two separate forms, each health personnel took the first set of measurements on all children before beginning the second set. This was intended to reduce recall of the first round values when taking second set of measurements. Completed forms from the first set were collected from observers before the second forms were distributed. Each health personnel recorded measurements independent of his or her paired colleague. For the purposes of this paper, the following measurements of reliability are of particular interest: technical error of measurement (TEM), average bias from expert, and coefficient of reliability (R) [17] (Equations for calculation of these measures of reliability are provided in the Additional file 1). TEM measures the error variability expressed in the same units as the measured variable. TEM can be intra-observer, using duplicate measurements by the same measurer, or inter-observer, where single measurements by two or more measurers are assessed [17, 18]. Average bias explains the average difference between measurements taken by an expert and those taken by measurers of the same subjects [17]. R, our main criterion to measure reliability, assesses the proportion of between-subject variance of measurements taken at one time that is free from measurement error [17].

### Case observations: measuring length at GMP clinic sessions

At community-based GMP clinics, an observation checklist, based on the training protocol, recorded the ability of all health personnel to measure length: positioning (child’s head touching headboard; legs lying flat and together on length board; feet perpendicular to base of length board), reading, and plotting on a WLZ chart. In addition, length of cases observed were measured first by health personnel (both nurses and health volunteers) and then by the observer and compared for agreement. At each of the ten GMP clinics, five cases per health personnel were observed. At each site, the mean GMP attendance of the community was divided by the quota (5 children per health personnel) to obtain a selection interval (k). The child being attended to, at the time of observer’s visit was identified as the first child. After that, every k^th^ child was selected to be measured until the quota was met. The case observation checklist was pretested in a non-participating community in the district before data collection.

### Interviews

One nurse and one health volunteer who had participated in all training sessions and used length measurements in their GMP clinics for a year were purposely selected from each community. Guiding questions used in interview covered health personnel’s perceptions on the difficulty or ease in using length measurements at outreach GMP, how measuring length affected their work in GMP, and the feasibility of implementing length measurements as part of GMP in the district. In addition, health personnel cited situations that prevented them from measuring length at GMP.

### Statistical analysis

We calculated intra- and inter-observer TEM, average bias from expert, and R from the standardization data. For our analysis, we selected the first measurement of each health personnel to compute inter-observer TEM. To calculate R, the standard deviation (SD) was obtained from length measurements of children 0–24 months in the UMKD population. The following approaches were used to judge acceptability of length measurements [18, 23]. Intra-observer TEM values for both nurses and health volunteers were considered adequate if they were within ±2 times of the expert’s Intra-TEM. For average bias from expert, a negative value indicated that the group of measurers underestimated the correct measurement whereas a positive value indicated an overestimation by the group measurement. This bias was considered large if differences between paired measurements exceeded 2.8 * intra-observer TEM of expert [17]. The R was used to estimate the proportion of inter-subject variance that was not due to measurement error. As a general rule consistent with that used for kappa, 0.8 was considered to be excellent agreement and 0.61 to 0.8 as good agreement [24].

Calculations were done with Excel 2007 and SPSS software - version 20 (SPSS Inc., Chicago Il., and USA). Results from observations are presented as means (SD) and percentages and the agreement of length measurements of health personnel and experienced anthropometrist, with the Bland Altman plot [25] and kappa. Qualitative data from interviews were audiotaped, transcribed verbatim, and evaluated via thematic analysis and coding.

## Results

### Reliability: standardization sessions

As expected for children under 24 months, the length measurements of children recruited for the standardization sessions ranged from 45 cm to 79 cm [26], Tables 2 and 3 show the intra-observer TEM and average bias from expert in both standardization sessions for each nurse and health volunteer. Based on the a priori accepted error standards, intra-observer TEM values for observers within ±2 times the expert’s TEM (that is, the expert’s 95% precision margin) were accepted [16, 17]. Intra-observer TEM for length measurements was considered acceptable in 14 out of 15 participants for both health volunteers and nurses at the first standardization session (session A). At session B, 14 nurses and 11 health volunteers out of a total of 15 in both groups met acceptable error standards for intra-observer TEM (Tables 2 and 3).

**Table 2:** Intra-observer and average bias of length measurements of community nurses by standardization session^a, b^

Nurse	Session A (*N* = 15)		Session B (*N* = 15)	
	Intra-observer TEM^a^	‖Average bias^b^	Intra-observer TEM^a^	Average bias^b^
A	0.87	−0.78	1.23	0.40
B	1.11	0.35	0.90	−0.20
C	1.22	0.38	0.95	−0.13
D	0.70	−0.95	0.72	−0.63
E	0.72	−1.33	0.96	−0.98
F	1.18	0.39	0.59	0.13
G	1.86	0.53	0.64	0.78
H	0.62	0.51	0.99	−0.15
I	2.77^c^	− 1.67	0.11	−0.65
J	0.68	−0.25	1.14	0.35
K	1.11	−0.83	0.11	−0.65
L	0.70	0.64	1.11	0.05
M	0.81	−0.63	0.63	0.68
N	0.41	−0.35	0.64	−0.10
O	1.33	−1.26	1.59^c^	0.07

^a^TEM: Technical error of measurement (Unit of TEM is cm)

^b^Average bias from expert anthropometrist

^c^Unacceptable according to error standard (2 * Intra-TEM of the expert’s)

‖Average bias for each community nurses met acceptable error standards (2.8 * Intra-TEM of the expert)

**Table 3:** Intra-observer TEM and average bias of length measurements of health volunteers by standardization session^a, b^

Volunteer	Session A (*N* = 15)		Session B (*N* = 15)	
	Intra-observer TEM^a^	‖Average bias^b^	Intra-observer TEM^a^	‖Average bias^b^
A	0.79	−0.07	0.63	−0.10
B	1.00	−0.22	0.63	−0.10
C	1.04	−0.47	0.97	−0.30
D	0.34	−0.40	0.10	−0.08
E	1.29	0.54	1.89^c^	− 0.28
F	0.97	0.08	0.67	0.75
G	1.85	0.54	0.61	0.62
H	1.13	−0.13	1.31	0.35
I	0.66	−0.15	1.12	−0.35
J	0.79	−0.63	2.50^c^	0.75
K	2.30^c^	0.28	0.64	− 0.08
L	0.97	0.38	1.95^c^	0.13
M	0.64	−0.60	1.16	0.45
N	1.11	−0.85	1.49^c^	− 0.03
O	1.12	0.11	0.60	−0.03

^a^TEM: Technical error of measurement (Unit of TEM is cm)

^b^Average bias from expert anthropometrist

^c^Unacceptable according to error standards (2 * Intra-TEM of the expert)

**‖**Average bias for each health volunteer met acceptable error standards (2.8 * Intra-TEM of the expert)

The intra-observer and inter-observer TEM, average bias from expert and R of length measurements taken at both standardization sessions for the group of nurses and group of health volunteers are shown in Table 4. Intra-observer TEM was comparable (1.07) in both groups at session A. However, intra-observer TEM was lower in the nurses group (0.82) compared to the health volunteer group (1.09) at session B. A similar trend was observed for the two groups of health personnel for inter-observer TEM. At session B, inter-observer TEM was 1.25 versus 1.59 in nurses and health volunteers, respectively. Inter-observer TEM was larger than intra-observer TEM for both groups at both sessions and was unacceptably (compared to error standards) high in both groups at both time points.

**Table 4:** Group intra-observer and inter-observer TEM, average bias and R of length measurements by standardization session and title ^a, b, c^

Session	Title	Intra- observer TEM^a^	Inter-observer TEM^a^	Average bias^b^	R^c^ (%)
A	Nurses	1.07	2.83	−0.348	60.3
	Health Volunteers	1.07	1.53	−0.104	88.5
B	Nurses	0.82	1.25	−0.07	92.3
	Health Volunteers	1.09	1.59	0.114	87.5

^a^Unit of TEM is cm

^b^Average bias from expert anthropometrist

^c^Coefficient of Reliability

The intra-TEM of the experienced anthropometrist was 0.7 cm for Session A, and 0.3 cm for Session B. The length measurements of all nurses and health volunteers met the accepted error standards for average bias from the expert at both time points. Generally, nurses and health volunteers tended to underestimate length measurements of the children (Tables 2 and 3). Based on average bias values, nurses tended to underestimate length measurements compared to experts at both sessions, although to a lesser extent at the later session. The health volunteer group slightly underestimated lengths at first session but a mixture of under- and over-estimation led to an overall positive sign for average bias (Tables 3 and 4) at session B. All biases in length measurements were within error limits of 2.8 times of the experienced measurer’s intra-observer TEM for both health personnel groups.

Coefficients of reliability for measurements by nurses were higher at session B compared to A, while that for health volunteers were not substantially different (Table 4). For both groups of health personnel, the proportion of inter-subject variance that was not due to measurement error, explained by R, ranged from good to very good agreement in both sessions [24]. At session A, nurses had about 40% of their length measurement variance compared to about 8% at session B, explained by measurement error. At both sessions, about 12% of the total variance in length measurements by health volunteers was explained by measurement error (Table 4).

### Observations

Analysis of characteristics of observed health personnel indicate that the mean (SD) years working in GMP was 3.6 (2.2) for nurses compared to 12.4 (9.6) for health volunteers. Nurses were mostly females (74%) while health volunteers were mostly male (74%). At GMP sessions, length measurements are typically taken together by a nurse with the help of a health volunteer. Thus, we were unable to separate ability to measure and plot by health personnel title.

**Fig. 1:**
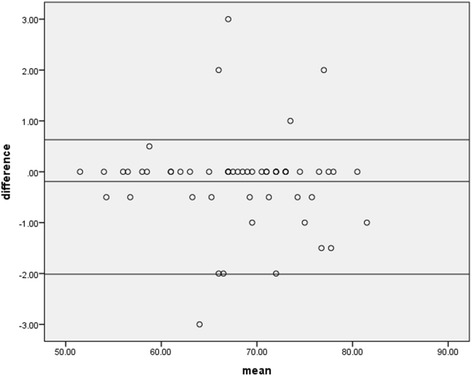
Comparing length measurements of health personnel to an experienced anthropometrist. A Bland-Altman plot showing level of agreement between length measurements taken by community health personnel and an experienced measurer at an outreach growth monitoring session in the Upper Manya Krobo district

In 51% of cases, health personnel met all three positioning techniques (child’s head touching headboard, legs lying flat and together on length board (with the exception of newborns and young infants), and feet perpendicular to base of length board) indicated in the training protocol. Accurate plotting was achieved in 67% (*N* = 50) of observed cases. Length measurements taken by health personnel and those taken by observer on observed cases were of moderate agreement (Fig. 1.; kappa = 0.53, *p* < 0.0001).

### Interviews

The interview data indicated that some community nurses and health volunteers experienced difficulty in adjusting to the use of length measurements. Child’s discomfort, as well as, mother’s sensitivity to baby’s crying were given as reasons for difficulty in adjusting to use of length measurements in GMP sessions.

“I don’t feel comfortable taking length measurements and the babies are also not comfortable. Trying to keep the child steady with all the crying really distresses the mother and me” (Health volunteer, UMKD).

However, frequent training improved confidence and skill in measuring length.

Nurses expressed a positive impact of refresher trainings on ability to use length tools (length measurements, plotting and interpretation) in GMP.

“I felt comfortable to measure length after three months of the initial training. After the initial training, I wasn’t confident in measuring length but the first refresher training helped me to get better” (Community nurse, UMKD).

Introducing length measurements into busy community-based clinics increased the duties of nurses who were responsible for providing several preventive services to both mothers and children during GMP. According to interviewed nurses, the introduction of length measurements affected usual GMP activities by increasing work load in communities with high GMP turnout.

“The biggest problem is that length measurements have extended GMP time. We have only two nurses in my health centre and one health volunteer assisting us. During GMP we also have to do immunizations, supplementation and attend to sick patients. Both the sick patients and mothers complain because we take longer than usual” (Community nurse, UMKD).

In such communities, when health volunteers did not show up at GMP sessions the workload for nurses was overwhelming. Health volunteers have no formal training in healthcare and were not compensated for their work. Thus, they were sometimes unavailable at GMP because of time conflicts with their primary occupation. With the introduction of length measurements and the new WLZ charts. Some health volunteers experienced difficulty in plotting.

“Health volunteers struggled with length measurements so very often, I had to leave my counseling desk to supervise plotting and make sure they were doing it right...” (Community nurse, UMKD).

Both groups of health personnel supported the introduction of length measurements into community GMP. However, there was a general perception that high workloads and few staff will be a significant barrier to long-term success.

“In my opinion I think it is possible but we need more hands, maybe more health volunteers or an extra nurse… or we will end up doing some shoddy work or not charting measurements properly. Everyone might think we are fully doing the measuring but the reality will be that we can’t handle the pressure. Extra hands, preferably nurse or well-trained volunteer, to help with measurement and other activities…” (Community nurse, UMKD).

## Discussion

The primary purpose of our study was to evaluate the reliability of length measurements collected by health personnel (both community nurses and health volunteers) working in outreach GMP at both standardization sessions and outreach GMP clinics. Precision and accuracy are equally important in standardized anthropometry training. In this study, Intra- and inter-TEM and R were used to measure precision and average bias measured the accuracy of length measurements collected by both health personnel groups. Intra- and inter-TEM identify the within- and between-measurer(s) error variability, respectively. R shows the percentage of total variability that is not due to measurement error and average bias points out how far the measurer(s) is from the “gold standard”. Three main observations can be made from our data. First, from an intra-observer TEM perspective and using error standards, length measurements by both nurse and health volunteer groups were reliable. The variations within measurer were lower at session B compared to A. On the other hand, the between measurer variations measured by inter-observer TEM were unacceptably high. Second, length measurements were within acceptable limits of average bias standard. However nurses tended to underestimate at both sessions while health volunteers underestimated at session one and overestimated length measurements in the later session. Third, we observed an improvement in reliability of nurses’ length measurements between the first and second sessions.

Published estimates for intra-observer TEM for length measurements range from 0.79–1.22 in newborns to 0.4–0.8 in young children (6–24 months) [18]. Ninety percent in Session A, compared to 70% in session B, of children used as subjects for reliability tests were within the 6 to 24 month age group. Only nurses’ group intra-TEM (0.82) in session B came close to those in the literature. Intra-TEM at session A for nurses and health volunteers’ group were higher than published estimates. However, care must be taken in making these comparisons. Number of observers, number of subjects, equipment and protocols used in training differ among studies. It is expected that inter-observer TEM (between-measurer variability) will be greater in magnitude than intra-observer TEM (within-measurer variability) [17]. However, wide variations between observers call for setting targets of precision that have to be achieved by nurses and health volunteers. The literature recommends the use of a criterion anthropometrist (selected based on consistent accuracy and precision of measurements) who can regularly oversee and assure the use of standard procedures and set targets for levels of precision and accuracy [17, 18].

Accuracy in standardized training refers to measuring length without bias [22]. The true value of the children’s length was unknown. Thus, the expert’s length measurement served as a “gold standard” and the average bias from expert gave an indication of accuracy of length measurements of nurses and health volunteers. Although the magnitude of the average bias fell within the allowable limits of the experienced measurer, distinct negative tendencies were noticed for both groups of health personnel. Length measurements of young children demand careful positioning to ensure that child is appropriately stretched before recumbent length is measured. Similar trends were found in the Multicentre Growth Reference study (average bias: − 0.70) to − 0.15) and the Rotterdam study standardization sessions (average bias: − 0.49 to − 0.15) where measurers, compared to experts underestimated length measurements. The average bias (− 0.35 to − 0.07) in this study was in the same direction and comparable to those in the above-stated studies. The average bias in the Multicentre Growth Reference study fell within the allowable limits of the experienced measurer and are reflected in the WHO charts [22, 27]. Thus, the comparable underestimations by nurses in this study are unlikely to affect the evaluation of wasting using the WHO WLZ or LAZ charts.

In general, standardization sessions are stressful for children, their mothers, and measurers as measurements often have to be repeated on crying, sometimes inconsolable, struggling children. Under such circumstances, an experienced measurer, has better control and self-assurance and is able to position children to full length before reading. In addition, an experienced measurer also has the advantage of being able to stretch legs of newborns adequately, while novices to length measurements are unsure and overly concerned about harming the baby. From our interviews with health personnel about their experience in using length as a GMP tool, child’s discomfort and mother’s sensitivity to baby’s cry was given as a reason for difficulty in adjusting to use of length measurements.

Considering our main criterion for assessing measurement reliability, the R of measurements for both groups ranged from moderate (60%) to very good (90%), although neither group reached the 95% cut off for length measurements recommended for research and surveillance purposes [16, 18]. At session B, nurses’ R (92.3%) came close to the acceptable cut-off (Table 4). Measurement errors are prone to changes over time. However, declines in anthropometry reliability can be prevented by ongoing training activities [16]. The knowledge and skill of community workers (a.k.a. Angawadi) on GMP activities was assessed in the Integrated Child Development Service in India. Frequent participatory in-service training was important in developing the anthropometric skill in growth monitoring community health personnel [28, 29]. In our study, we conducted refresher trainings on length measurements before both sessions A and B and at the midpoint between the two sessions (three months after session A). Perhaps, more frequent training sessions are needed for new measurers. Nurses expressed a positive impact of refresher trainings on their ability to use length tools in GMP. The reliability of length measurements improved in Session B (after two refresher trainings) for nurses but not for HV. In this study, training of some HV to measure and plot length measurements was challenging. While all nurses had completed a two-year post-secondary diploma in community nursing, the level of education of HV ranged from mostly some primary to a few post-secondary years. In addition, the GHS’s HV position is unpaid. The primary occupations of HV often prevented them from being available for GMP sessions, ridding them of opportunities of practicing the skill of collecting lengths. It is difficult to compare measurements collected during standardization and GMP sessions because the settings are different. In the former, health personnel had to collect duplicate measurements on 10 children and were not allowed to compare measurements and take new measurements. At GMP, health personnel collected one measurement, as typically done for weights, and dealt with children one at a time. However, kappa, a measure of agreement comparable with R indicated moderate reliability (0.53, *p* < 0.0001) of GMP length measurements. Differences at GMP and standardization sessions could be attributed to overburdening tasks, exhaustion, and the pressure on nurses and health volunteers to attend to all waiting mothers and their children on the GMP clinic day. Since GMP sessions are held once monthly, turn out in larger communities can be overwhelming for nurses and health volunteers. Half of the observed health personnel failed to meet all positioning recommendation (child’s head touching headboard, legs lying flat and together on length board (with the exception of newborns and young infants), and feet perpendicular to base of length board) in the training protocol. Ulijaszek and colleagues recommend the prevention of tiredness in personnel taking measurement as this can lead to bias in length measurements. According to interviewed nurses, the introduction of length measurements affected usual GMP activities by increasing work load in communities with high GMP turnout. When health volunteers missed sessions in these communities, nurses were overwhelmed with their duties.

Accurate plotting was observed in 67% of observed cases. In most GMP settings, health volunteers measure and plot, while nurses provide health and nutrition counseling, immunizations, deworming, and vitamin A supplementation. Unlike nurses, health volunteers typically do not have any formal training in health care. With the introduction of length measurements and WLZ charts. Some health volunteers experienced difficulty in plotting, this could be explained by poor eyesight complaints from health volunteers during training sessions.

In light of these challenges, nurses and health volunteers were asked about their perception of the feasibility of using length measurements as part of routine GMP activities. Although nurses were generally positive about the feasibility of implementation, they perceived that its success was dependent on the provision of adequately trained staff to assist with multiple duties.

Our results indicate that the ability of community nurses and health volunteers to collect reliable length measurements is promising. This is an important finding given the recommendation by the WHO for developing countries to introduce length as part of GMP programs [2], and the concerns by local programs whether community-based health personnel can measure length reliably. Specifically, the study contributes to the knowledge on both individual and institutional barriers to the collection of precise and accurate length measurements in a rural community-based GMP program. The reliability of length measurements taken during GMP sessions may be more susceptible to errors due to overburdened personnel and understaffed GMP clinics. Thus, the issue of prime import is to review the personnel insufficiency experienced in rural community-based GMP and make the necessary adjustments alongside the introduction of length measurements. Next, imprecision and inaccuracy can be minimized by regular in-service training. Calibration checks, regular duplicate measurements in a subgroup of children attending GMP can help to establish reliability of length measurements and improve accuracy. In addition, selecting criterion anthropometrists [17] in defined localities, who can oversee and assure the use of standard procedures and set targets for levels of accuracy and precision that new and existing measurers could aim to achieve is essential for ensuring the quality of length measurements over time.

In summary, the potential exists for reliable length measurements to be included in regular community-based GMP activities. However there are a number of issues that need to be addressed to make this successful and sustainable. Well-trained health personnel are needed to assist in delivery of GMP services. Nurses must receive pre-service training to collect both accurate and precise length measurements in nursing school and health volunteers must be trained before assisting in the community. This will help build skill and confidence to measure accurately and precisely before working in the community clinics. Nurses and health volunteers who are already working in the communities must receive several in-service trainings to improve skill. During training, attention should be paid to between- measurer error variability as they are likely to be unacceptably high in these groups of health personnel. Other useful considerations for GMP include the working environment for data collection. Measurement stations should be planned so that there is adequate space to prevent additional error as a consequence of crowding, misrecording, or both at GMP clinics. In addition, positioning of length board on leveled surfaces is important to achieve accurate measurements. The introduction of length measurements at GMP will aid monitoring of stunting in children. Nurses must be trained to provide effective counseling for mothers of “stunting” children. There is a need to design messages that address the multifactorial determinants of stunting.

To ensure the success of implementing length measurements as part of community-based standard-of-care GMP activities, issues of understaffing of nurses and resource constraints need to be addressed by the GHS and pre-service and in-service training provided for health personnel in GMP. Considering the essential role played by health volunteers in community-based GMP, it is recommended that the GHS appraises the use of volunteers and encourage the selection of competent volunteers by local community heads, who can fully participate in health activities of their communities. Although it is beyond the scope of this paper, we recommend that supportive supervision and mechanisms to motivate both nurses and health volunteers be developed to encourage high performance.

## Conclusions

Our findings show that the potential for community nurses and health volunteers in rural outreach GMP services to collect reliable length measurements is promising. However, the reliability of length measurements taken during GMP sessions may be susceptible to errors due to lack of confidence in the skill of measuring length, overburdened health personnel, and crowded GMP clinics. There is need for both pre- and in-service training of nurses and HV on length measurements and measures to improve and maintain reliability of length measurements.

## Additional file

Additional file 1: Appendix: Equations used in estimating reliability. Eq. 1 for calculating Intra-observer TEM, Eq. 2. for Inter-observer TEM, Eq. 3. for Average bias from the expert, and Eq. 4. for the calculation of Coefficient of reliability (R) (DOCX 20 kb)

## Abbreviations

GHS: Ghana Health Service
GMP: Growth Monitoring and Promotion
HAZ: Height-for-age Z score
HV: Health Volunteer
LAZ: Length-for-age Z score
R: Coefficient of Reliability
SD: Standard Deviation
SPSS: Statistical Software for Social Sciences
TEM: Technical Error of Measurement
UMKD: Upper Manya Krobo District
WAZ: Weight-for-Age Z score
WHO: World Health Organization
WHZ: Weight-for-Height Z score
WLZ: Weight-for-Length Z score
