# Supplementary material for: Reliability of length measurements collected by community nurses and health volunteers in rural growth monitoring and promotion services
Source: BMC Health Serv Res. 2018 Feb 17;18:118. doi: 10.1186/s12913-018-2909-0 (PMC5816548; doi:10.1186/s12913-018-2909-0)
Supplement: Supplementary file 1 — Appendix: Equations used in estimating reliability. Eq. 1 for calculating Intra-observer TEM, Eq. 2. for Inter-observer TEM, Eq. 3. for Average bias from the expert, and Eq. 4. for the calculation of Coefficient of reliability (R) (DOCX 20 kb) [file 12913_2018_2909_MOESM1_ESM.docx]

## Appendix

Equations used in estimating reliability [17]:

**Equation 1. Intra-Observer TEM**

Calculated for multiple measurements made by multiple observers on the same subject.

where *K* = number of observers

*M_ij1_ & M_ij2_* = duplicate readings recorded by observer j for the ith child

*N_j_* = number of children measured by observer j

**Equation 2. Inter-observer TEM**

Calculated for one measurement of a subject made by multiple observers.

$$\left\{\frac{1}{N}\sum_{i=1}^{N} \frac{1}{(K_{i}-1)}\left[\sum_{j=1}^{K_{i}} Y_{ij}^{2}-\frac{(\sum_{j=1}^{K_{i}} Y_{ij})^{2}}{K_{i}}\right]\right\}^{\frac{1}{2}}$$

where *K_i_ =* Number of observers that measured child i

*N =* Number of children involved,

*Y_ij_ =* One of the duplicate measurements taken by observer j for the ith child

**Equation 3. Average bias from the expert**

Calculated as the average difference between measurements taken by an expert and those taken by observers of the same subjects [17].

$$\frac{\sum_{i=1}^{N_{G}} \left[\sum_{j=1}^{K} \left(M_{ij1}+M_{ij2}\right)/\left(2*K\right)-\left(M_{iG1}+M_{iG2}\right)/2\right]}{N_{G}}$$

where *K =* Number of observers measuring the same children

*M_ij1_ & M_ij2_* = duplicate readings recorded by observer j for the ith child

*M_iG1_ & M_iG2_ =* duplicate readings recorded by expert for the ith child

*N_G_ =* Number of children measured by the expert

A negative value means that the group underestimates the correct measurement and a positive value means group overestimates measurement. The bias was considered large if it exceeded (2.8 * intra-observer TEM of expert)[17].

**Equation 4. Coefficient of reliability (R)**

R estimates proportion of inter-subject variance that is not due to measurement error. As a general rule, 0.8 was considered to be excellent agreement and 0.61 to 0.8 as substantial agreement.

$$1-\frac{\left(TEM\left(Inter\right)\right)^{2}}{SD^{2}}$$

where *SD* = Standard deviation

*TEM (Inter)* = Inter observer TEM
